# Multi-month prescriptions, fast-track refills, and community ART groups: results from a process evaluation in Malawi on using differentiated models of care to achieve national HIV treatment goals

**DOI:** 10.7448/IAS.20.5.21650

**Published:** 2017-07-21

**Authors:** Margaret L. Prust, Clement K. Banda, Rose Nyirenda, Frank Chimbwandira, Thokozani Kalua, Andreas Jahn, Michael Eliya, Katie Callahan, Peter Ehrenkranz, Marta R. Prescott, Elizabeth A. McCarthy, Elya Tagar, Andrews Gunda

**Affiliations:** ^a^ Applied Analytics Team, Clinton Health Access Initiative, Inc., Boston, MA, USA; ^b^ Malawi Country Team, Clinton Health Access Initiative, Inc., Lilongwe, Malawi; ^c^ Department of HIV and AIDS, Ministry of Health, Lilongwe, Malawi; ^d^ I-TECH, Department of Global Health, University of Washington, Seattle, WA, USA; ^e^ HIV, TB and Health Financing Team, Clinton Health Access Initiative, Inc., New York, NY, USA; ^f^ Bill and Melinda Gates Foundation, Seattle, WA, USA; ^g^ Applied Analytics Team, Clinton Health Access Initiative, Inc., Lusaka, Zambia; ^h^ HIV, TB and Health Financing Team, Clinton Health Access Initiative, Inc., Melbourne, Australia

**Keywords:** HIV, differentiated care, process evaluation, costing, mixed methods

## Abstract

**Introduction**: In order to facilitate scale-up of antiretroviral therapy (ART) in Malawi, innovative and pragmatic models have been developed to optimize the efficiency of HIV service delivery. In particular, three models of differentiated care have emerged for stable patients: adjusted appointment spacing through multi-month scripting (MMS); fast-track drug refills (FTRs) on alternating visits; and community ART groups (CAGs) where group members rotate in collecting medications at the facility for all members. This study aimed to assess the extent to which ART patients in Malawi are differentiated based on clinical stability and describe the characteristics and costs associated with the models of differentiated care offered.

**Methods**: A mixed methods process evaluation was conducted from 30 purposefully selected ART facilities. Cross-sectional data for this evaluation was collected between February and May 2016. The following forms of data collection are reported here: structured surveys with 136 health care workers; reviews of 75,364 patient clinical records; 714 observations of visit time and flow; and 30 questionnaires on facility characteristics.

**Results**: Among ART patients, 77.5% (95% confidence interval [CI] 74.1–80.6) were eligible for differentiated models of care based on criteria for clinical stability from national guidelines. Across all facilities, 69% of patients were receiving MMS. In facilities offering FTRs and CAGs, 67% and 6% of patients were enrolled in the models, respectively. However, eligibility criteria were used inconsistently: 72.9% (95% CI 66.3–78.6) of eligible patients and 42.3% (95% CI 33.1–52.0) ineligible patients received MMS. Results indicated that patient travel and time costs were reduced by 67%, and the unit costs of ART service delivery through the MMS, FTR and CAG models were similar, representing a reduction of approximately 10% in the annual unit cost of providing care to stable patients that receive no model.

**Conclusions**: MMS is being implemented nationally and has already generated cost savings and efficiencies in Malawi for patients and the health system, but could be improved by more accurate patient differentiation. While expanding FTRs and CAGs may not offer significant further cost savings in Malawi, future studies should investigate if such alternative models lead to improvements in patient satisfaction or clinical outcomes that might justify their implementation.

## Introduction

Malawi has achieved significant expansion of ART coverage in recent years. Of the estimated 979,000 HIV-positive individuals in Malawi in March 2016, 62% were on ART [1]. Coverage must continue to expand in order to meet the UNAIDS 90–90-90 goals for 2020 that aim to ensure 90% of all people living with HIV know their HIV status, 90% of all people with diagnosed HIV infection receive sustained ART, and 90% of all people receiving ART have viral suppression. To support these goals, the World Health Organization (WHO) released ART guidelines recommending a “treat-all” approach, whereby all HIV-positive populations and age groups are eligible for ART [2]. In line with this recommendation, Malawi launched revised ART guidelines in August 2016 [3]. While these revised guidelines pave the way for the country to achieve its ambitious treatment targets, Malawi faces significant financial and human resource constraints. UNAIDS estimates a national HIV/AIDS funding gap of at least 50% through 2022–2023 [4].

Malawi has a history of adopting a pragmatic approach to scaling up HIV care and treatment through innovation. For example, Malawi was the first country to simplify delivery of prevention of mother-to-child transmission (PMTCT) services by rolling out what became known as “Option B+” [5]. Now, a variety of facility- and community-based models of differentiated care have been piloted in Malawi to categorize adult ART patients and adjust service delivery to patient needs. Prior to implementation of differentiated care, most ART patients made the same number of visits and saw the same cadre of healthcare workers at health facilities regardless of their disease progression. Since a large proportion of ART patients are stable and healthy, providing streamlined models of differentiated care for these patients may offer opportunities to improve service delivery efficiency while also maintaining quality [6,7].

Three promising, complimentary models of differentiated care are being implemented in Malawi and were prioritized by the Ministry of Health (MOH) and other stakeholders for further understanding through this analysis. These included two facility-based models focused on individual clients, multi-month scripting (MMS) and fast-track refills (FTR), as well as one group-based, community-level model, community ART groups (CAGs). For MMS, which has been part of the national ART guidelines in Malawi since 2008, stable patients are given three-month rather than one-month refills, reducing the number of facility visits to four per year for stable patients. In addition to MMS, some facilities are offering either the FTR model or the CAG model, but we identified no sites that are currently offering FTRs and CAGs. In the FTR model, stable patients still make four facility visits every year, but only two visits are standard clinical visits with a clinician. The other two visits are less time- and resource-intensive refill visits during which drug refills are provided by a lower-level health surveillance assistant (HSA). CAGs are peer-led groups that meet at the community level for ARV distribution and peer-led discussions. For each appointment, a different group member collects ARVs for the entire group. These models can be considered in terms of the adjustments made to visit frequency (when), service intensity (what), cadre (who), and service location (where) [8] (Table 1).

**Table 1.** Characteristics of key models of differentiated care^a^

	WHEN	WHAT	WHO	WHERE
Model of care	Visit frequency	Service intensity	Cadre	Location
Standard of care (for patients that are not eligible for or accessing any differentiated models of care)	Monthly	Clinical consultation, individual counseling, and drug refills offered at each visit	All consultation services provided by a nurse or clinician; dispensing by pharmacy or other skilled staff	All services are facility-based
Multi-month scripting (MMS)	Eligible patients go to clinic every three months (four clinical consultations per year)	Clinical consultation, individual counseling, and drug refills offered at each visit	All consultation services provided by a nurse or clinician; dispensing by pharmacy or other skilled staff	All services are facility-based
Fast-track refills (FTRs)^a^	Eligible patients go to clinic every three months (two clinical consultations and two drug refill visits per year)	Clinical consultation, offered every six months; interim visits are for drug refills only without weighing, health talk or full consultation	All consultation services provided by a nurse or clinician; drug refill visits managed by a health surveillance assistant (HSA) instead of nurse or pharmacy staff^b^	All services are facility-based
Community ART groups (CAGs)^c^	One member of group goes to clinic every month, individual patients go to clinic approximately every 6 months	Clinical consultation and individual counseling offered to the member representing the group for that visit	All facility-based consultation services provided by nurse or clinician to the group representative; regular monitoring checks by group members, with community monitoring from an HSA^b^	Recording of patient information and medication dispensing happens at community meetings

^a^More information about the FTR model being implemented in Malawi with support from MSF-France (under the name six-month appointment [SMA]) is available from external sources [9].

^b^In the case of both FTRs and CAGs, medication dispensing is handled in part by HSAs or by community members. Although this approach has been piloted in Malawi, official policies continue to state that only a nurse or high-level health worker can dispense HIV medications.

^c^More information about the CAG model is available through MSF-Belgium. In particular, refer to the CAG toolkit available from: http://samumsf.org/documents/2015/01/cag-toolkit.pdf.

This evaluation was conceived in response to policy makers’ demand for evidence to evaluate existing models of differentiated care and refine Malawi’s service delivery guidelines. There was limited coordination in implementation across facilities and partners and limited evidence available. Differentiated models of care are needed to meet goals to expand access to HIV treatment. We sought to understand how best to implement these models in a real-world clinical setting in Malawi. Specifically, this process evaluation sought to define the existing models, understand the extent to which patients in Malawi were differentiated according to clinical stability criteria, and analyse the characteristics and costs of the models of differentiated care they received. While there are additional outcomes that may be influenced by these models, such as clinical outcomes and patient satisfaction, the focus of this work was particularly on the accuracy of differentiation of patients for the models and on the costs and potential cost savings generated by the models. It was not within the scope of this study to assess the clinical outcomes associated with patients in the three models.

## Methods

We conducted a mixed methods process evaluation in 30 purposefully-selected facilities. Specifically, we used heterogeneity sampling to achieve diversity in the sample based on: model of differentiated care offered, region, patient volume, facility owner (i.e., MOH or Christian Health Association of Malawi [CHAM]), facility type, and implementing partner support. Data was collected between February and May 2016 by two teams of five data collectors. Data collection methods included: interviews with ART in-charges, patient focus groups, health worker surveys, visit time observations, patient record review, and facility information questionnaires. Results from interviews and focus groups are not reported in this paper. All paper-based data forms were entered using EpiData and random selection of 25% of data was double-entered. Analysis was performed using Stata 13.

### Health worker surveys

A survey was orally administered to health workers to understand the challenges faced by and recommendations of health workers relating to the implementation of models of differentiated care. All health workers involved with HIV care in all 30 facilities in the sample were invited to participate.

### Visit length and costing

Visits were observed to understand how much time patients spent waiting and being serviced under each model of care and the cadre of health worker providing each service. *A sample size of 26 observations was required from each site in order to obtain an average visit length with accuracy of ±10 min and a confidence of 0.05* [10].

Patients were tracked throughout their entire visit beginning at the time of the first service encounter. In order to minimize interference with service provision, there was no interaction between data collectors and patients. Descriptive statistics were estimated including the average wait and service station times in various facility types. Modelling was performed to identify the unit costs of each model of care using current government salary scales to estimate the cost of health worker time along with other cost assumptions derived from previous facility-based costing studies conducted in Malawi by CHAI and MOH [11]. (Additional details about the costing assumptions are available in the supplementary material.)

### Patient record reviews

Patient records were used to assess the extent of implementation of each model of care and the accuracy of patient differentiation. The sample included adult patients (18 years or older) that were active in care at the time of data collection. For 17 sites using electronic patient records managed by Baobab Health Trust or Médecins Sans Frontières (MSF) France, all active, adult patients were included in the sample. For the 13 sites using paper-based records, we randomly sampled patients. Assuming that 50% of patients receive the models, we determined a sample size of 24 patients per facility was required to estimate the percentage of eligible patients receiving the models of care with a precision of 20% and confidence of 0.05 [10]. Assuming conservatively that only 20% of patients would be eligible and that an additional 20% would be missing, we required a sample of 151 patient records to achieve the final desired sample of 24 patients per facility. Results were weighted to adjust for the differential sampling proportions across sites and clustered to adjust for similarities between patients from the same facility.

The criteria shown in Table 2 were expected to be used by health care workers to determine patient eligibility for enrolment in one of the alternative models of care.

**Table 2.** Criteria to be considered eligible for differentiated models of care

Criteria component	Eligibility criteria for MMS^a^	Eligibility criteria for FTRs and CAGs^b^
Age	18 or older	18 or older
Amount of time on ART	At least six months on ART	At least six months on ART
Regimen	On a first line regimen ^c^	On a first line regimen
Side effects	No adverse drug reactions	No adverse drug reactions
Opportunistic infections (OIs)	No current illnesses or OIs	No current illnesses or OIs
Viral load (VL) testing^d^	VL less than 1000 copies/ml	VL less than 1000 copies/ml
Adherence	Good adherence	Good adherence
Pregnancy	–	Not pregnant or lactating^e^

^a^Stable and adherent patients have been eligible for three-month refills in Malawi since the 2008 National Guidelines on the Use of Antiretroviral Therapy in Malawi. Upon consultation with the MOH, these criteria were given as the specific characteristics that health workers are trained to use to determine eligibility for MMS.

^b^Criteria for FTRs and CAGs were developed by MSF-France and MSF-Belgium, respectively, in collaboration with the MOH. Because these are not national programs, these criteria are not included in the national ART guidelines.

^c^Under the MOH guidelines, patients on second-line therapy and meeting other criteria may be eligible for 2-month refills, but the criteria listed above are for 3-month refills.

^d^Viral load testing is currently being scaled up in Malawi but not all patients had received a viral load test at the time of data collection. In the event of missing viral load data, health workers determined eligibility using data available on the other criteria contained in this table.

^e^Pregnant and lactation women can remain social members of the CAG in the sense that they meet with the group, but they have to go to the facility more frequently than other CAG members to receive ANC and PMTCT services.

### Facility information questionnaire

We collected data about facility-level characteristics that may influence implementation of models of care using a structured questionnaire. The questionnaire assessed information on urbanicity, catchment population, staffing, and laboratory testing protocols. The data collection teams consulted with the ART in-charge to complete the questionnaire.

## Results

The characteristics of the sampled facilities are shown in Table 3. The characteristics of patients in the final sample for each data collection method are shown in Table 4. Overall, we surveyed 136 health workers (out of 161 health workers invited to participate); reviewed 75,364 patient records; conducted 714 observations of visit time and client flow; and completed 30 facility questionnaires. Our data entry error rate ranged from 0.7% (health worker surveys) to 5.0% (visit time).

**Table 3.** Facility characteristics

Characteristic	*N*	% of facilities
**Facility type**		
Hospital	15	50.0
Health centre or clinic	15	50.0
**Urbanicity**		
Rural or remote	16	53.3
Urban	14	46.7
**Active ART patients**		
Less than 500	3	10.0
501–2000	9	30.0
2001–6000	15	50.0
More than 6000	3	10.0
**Differentiated models of care offered**		
MMS	30	100.0
FTR	4	13.3
CAG	8	26.7

**Table 4.** Characteristics of study participants, by data collection method

Data collection tool/characteristic	n^a^	(%)
**Health worker survey**	**136**	
*Cadre*		
Clinical Officer	9	6.6
Nurse or Nurse Midwife Technician	44	32.4
Health Surveillance Assistant	22	16.2
Data Clerk	33	24.3
Other^b^	28	20.6
Years in role (mean, SD)	5.9 (4.5)	
**Patient record reviews**	**75,364**	
*Data source*		
Paper-based records	1857	2.5
MSF-France electronic records	13,388	17.8
Baobab Health Trust electronic records	60,119	79.8
*Gender* (weighted and clustered %, 95% CI) ^c^		
Male	25,850	34.3 (32.6 – 36.1)
Female	49,514	65.7 (63.9 – 67.4)
Age (mean, SE)	40.4 (0.6)	
Years in ART care (mean, SE)	4.5 (0.2)	
**Visit time observations**	**714**	
**Gender**		
Male	216	31.6
Female	467	68.4
**Age^d^**		
18 to 30	85	12.6
31 to 45	330	48.9
46 to 64	250	37.0
65 and over	10	1.5
**Visit type^e^**		
Standard site – All patients	404	56.6
FTR site – HSA refill	44	6.2
FTR site – Standard visit	60	8.4
CAG site – CAG member	17	2.4
CAG site – Non-CAG member	189	26.5

^a^Numbers may not sum to total due to missing data.

^b^For the health worker survey, other includes small representations among the following cadres: HIV Diagnostic Assistants, clinic aides, lay volunteers, medical assistants, counsellors, hospital attendants, and unspecified.

^c^Figures on the number of men and women represented in the sample are estimated based on weighted and clustered proportions.

^d^For the visit time observations, age and gender were estimated by the data collector without patient interaction.

^e^Visit type was determined after data collection based on stations visited.

### Description and coverage of models of care

In March 2016, there were 608,028 patients on ART across 730 ART sites nationally [1]. While MMS was offered in all 730 facilities, only 5.4% and 9.4% of patients attended facilities where the FTR and CAG models were offered, respectively (Table 5). The FTR model has been implemented since 2008 with support from MSF-France in Chiradzulu district, where it was referred to as the “six-month appointment” program. The term SMA refers to the fact that patients only have a full clinical consultation every six months, but should not be confused with the provision of six-month refills. The CAG model was offered in Thyolo district since 2012 and in Nsanje district since 2015 with initial support from MSF-Belgium and currently through the leadership of the district health offices. Based on patient record review data and among patients receiving care from a facility offering each model, approximately two-thirds of patients in MMS and FTR facilities participated in the model. However, only 6.0% of patients in facilities that offered the CAG model were enrolled in a CAG. Participation was similar in Thyolo district where CAGs were launched in 2012 (4.4%, 95% CI 0.4–34.0) and in Nsanje district where CAGs were launched in 2015 (11.9%, 95% CI 2.4–43.0). There were no significant differences in the distribution of male and female participants in each model.

**Table 5.** Access to and participation in key differentiated models of care

Model of care	Multi-month scripting	Fast-track refills	Community ART groups
Active patients with access to model^a^ (*n*, %)	608,028 (100.0)	32,682 (5.4)	57,408 (9.4)
Sites offering model (*n*, %)	730 (100.0)	11 (1.5)	34 (4.7)
Proportion of patients participating in the model (within facilities offering the model) (95% CI)^b^	68.7 (62.5–74.6)	66.8 (59.4–73.5)	6.0 (0.9–30.6)
Proportion of *males* participating in the model (within facilities offering the model) 95% CI)^b^	71.2 (64.9–76.7)	69.0 (60.5–76.5)	5.0 (0.6–33.3)
Proportion of *females* participating in the model (within facilities offering the model) (95% CI)^b^	67.5 (61.1–73.2)	65.8 (58.1–72.7)	6.4 (1.1–29.7)

^a^Access to the model indicates that the patient attends a facility where the model is offered, regardless of that patient’s clinical stability. Facility patient volumes are based on MOH progress reporting as of January 2016 [1].

^b^Results are based on patient record reviews and are weighted and clustered.

### Patient differentiation

Based on patient record reviews, the percentage of patients excluded from differentiated models of care on the basis of each individual criterion is shown in Table 6. For purposes of this evaluation, we assumed patients were eligible unless otherwise specified. Patients with missing data for a characteristic were not considered ineligible based on that characteristic.

**Table 6.** Percentage of adult patients categorized as not eligible for differentiated care or with missing data for each individual inclusion criteria (weighted and clustered results)a

Inclusion criteria	% not eligible^b^(95%CI)	% unclear or missing data (95% CI)
On ART less than six months	7.5 (5.8–9.5)	5.7 (2.0–15.7)
On second- or third-line ART	2.5 (1.6–3.8)	0.0 (0.0–0.1)
Reporting adverse drug reactions	0.6 (0.2–1.3)	27.5 (14.2–46.5)
With confirmed or suspected TB^c^	0.8 (0.5–1.2)	7.2 (3.1–16.0)
Higher than or equal to 1000 copies/ml	6.2 (3.6–10–9)	69.7 (51.2–83.4)
Poor adherence^d^	0.0	100.0
Pregnant or lactating^e^	10.9 (8.8–13.4)	29.0 (21.8–37.6)

^a^This table examines eligibility on individual criteria only. Patients may be ineligible on the basis of multiple criteria though. Age was used as an exclusion criterion for the overall study, therefore all data collected was related to patients over the age of 18 and that criterion is not listed here.

^b^Represents only patients who were confirmed by chart review to not meet one of the inclusion criteria for clinical stability.

^c^The inclusion criteria specify that patients should have “No current illnesses or OIs,” but data was only available about TB.

^d^For Baobab and paper-based records, information was collected about the number of doses missed since the last visits, but the data was found to be inconsistent and was therefore not used. No adherence data is recorded in MSF-France sites.

^e^For patients with paper-based records, pregnancy status is only available at the time of enrolment but not at the last visit. Lactation data is not available for MSF-France sites. All males are recorded as being not pregnant or lactating.

Considering all of the criteria, 77.5% (95% CI, 74.1–80.6) of patients from this sample were eligible for FTRs and CAGs, and 86.4% (95% CI, 84.0–88.6) were eligible for MMS. However, this estimate of the eligible population is likely higher than the true proportion, because this analysis does not account for patient adherence due to inconsistencies in the data about the number of doses missed since the last visit across record types.

In interviews, ART in-charges seemed to have a generally high level of understanding of the eligibility criteria, but data from health worker surveys revealed that other health workers in the facilities may have a less comprehensive understanding of the inclusion criteria. Specifically, an open-ended survey question was used to ask health workers about the criteria used to establish patient eligibility. Among nurses and clinicians, who are responsible for determining model eligibility and should be most aware of the criteria, adherence (72%) and length of time on ART (62%) were most often mentioned. But even among nurses and clinicians, some criteria, such as ARV regimen (2%), pregnancy status (10%), and age (16%), were rarely cited.

Using patient record reviews, we assessed the degree to which patients were being accurately classified, based on the relevant guidelines. Based on the MOH criteria for MMS, we found that among all patients that were eligible at their most recent visit, 72.9% received MMS at that visit (Table 7). Based on the FTR and CAG criteria, 77.7% of eligible patients at facilities offering the model were enrolled in the FTR program, and 6.0% were enrolled in a CAG. Among ineligible patients in facilities offering each model, 42.3% received MMS at their last appointment, 31.4% were enrolled in the FTR programme, and 5.6% were enrolled in a CAG.

**Table 7.** Patient eligibility and participation in differentiated models of care (weighted and clustered results)^a^

Differentiated model of care	Eligible patients	Ineligible patients
**MMS**		
Percentage receiving MMS	72.9 (66.3–78.6)	42.3 (33.1–52.0)
Percentage not receiving MMS	27.1 (21.4–33.7)	57.7 (48.0–66.9)
**FTRs**		
Percentage enrolled in FTRs	77.7 (66.1–86.1)	31.4 (24.7–38.9)
Percentage not enrolled in FTRs	22.2 (12.9–33.9)	68.6 (61.1–725.3)
**CAGs**		
Percentage enrolled in CAGs	6.0 (0.9–31.0)	5.6 (0.9–28.3)
Percentage not enrolled in CAGs	94.0 (69.1–99.1)	94.4 (71.7–99.1)

^a^For this table, the criteria relevant to each model of care are used to determine which patients are eligible. Specifically, pregnant and lactating women are considered eligible for MMS.

**Figure 1.**
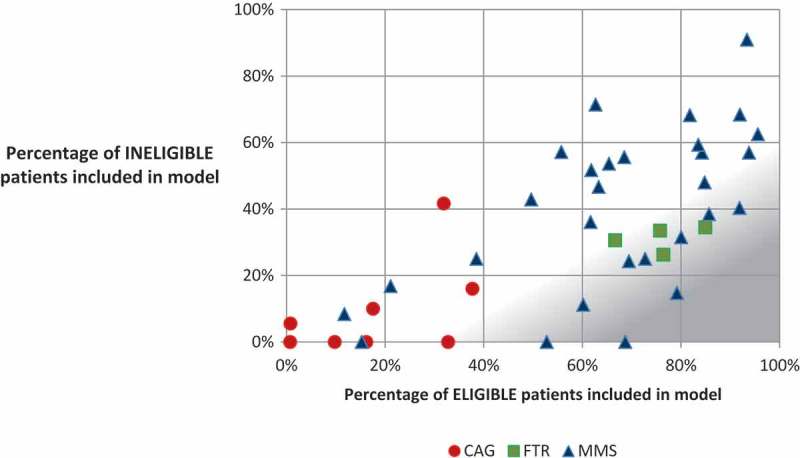
Percentage of eligible and ineligible patients included in differentiated models of care, by facility. ^1^The area shaded in grey represents more optimal patient differentiation, where a high percentage of eligible patients and a low percentage of ineligible patients are included in the model.

In individual facilities, there was variation in the application of eligibility criteria, particularly in relation to MMS (Figure 1). For example, some facilities were successful in ensuring that ineligible patients did not receive MMS, but those same facilities often provided few eligible patients with MMS, meaning that the facilities were not optimally benefitting from the efficiency gains of MMS. On the other hand, other facilities provided MMS to a large percentage of eligible patients, but also provided MMS to a large percentage of ineligible patients, meaning that ineligible patients may not have received the care recommended in national guidelines. Several facilities performed particularly well in terms of accurate patient differentiation. Upon inspection, these facilities represented relatively small facilities that have few ineligible patients. Still, those facilities seem to be performing well in terms of identifying cases for referral to another facility or more intensive care within the same facility. In general, facilities offering FTRs enrolled a consistently high percentage of eligible patients and a relatively low percentage of ineligible patients in FTRs. Facilities offering CAGs were consistent in enrolling a relatively low percentage of ineligible patients in CAGs, but the enrolment of eligible patients was also lower than that of other models.

Survey data and staff interviews provided the following reasons why eligible patients may not be included in the models: lack of health worker knowledge of criteria or not recognizing when patients are eligible; stockouts or low stocks preventing MMS from being feasible; patient preferences (specifically in the case of CAGs). Ineligible patients may be included in the models due to: lack of understanding of eligibility criteria by health workers; special requests from patients to participate; health worker desire to reduce workload or congestion; patients becoming ineligible and being transferred out of FTR or to be a social member of a CAG (participating in group meetings but not in the medication dispensing aspect of the group); and unrecognized transitions of patients from eligible to ineligible.

### Costs and burden of care

We assessed variations in the costs of care – both for facilities and patients – by combining data about the frequency, cadre, intensity and location of services. On average, an eligible patient in a standard facility not offering FTRs or CAGs spends 3.0 h at the facility for each appointment, of which 2.2 h is spend waiting and 0.8 h is spent receiving services. Patient time and travel costs are reduced by all three models of care, compared to a patient that is receiving no models (Table 8), with the time requirements being lowest for FTRs and the travel costs being lowest for CAGs.

**Table 8.** Average patient time and cost summary, by visit type^a^

Site and visit type	Visits per year	Travel time per visit (min)^b^	Wait time per visit (min)^c^	Servicing time per visit (min)^c^	Total time per year (h)	Total travel cost per year (MWK)
Patient receiving no models	12	193.8	131.2	49.2	74.8	4,972
Patient receiving MMS	4	193.8	131.2	49.2	24.9	1,657
Patient enrolled in FTR and receiving MMS^d^	4	193.8	93.0	26.5	20.9	1,657
Patient enrolled in CAG – community level meetings^e^	12	0.0	0.0	120.0	36.8	829
Patient enrolled in CAG – facility level visits	2	193.8	131.2	59.5		

^a^This table represents visit times for the typical, eligible patient and does not include extra care provided to ineligible patients. Also, this table does not represent wait or servicing times for laboratory visits, which occur every two years.

^b^ Travel time represents the average round-trip travel time reported by 216 participants in patient focus groups. Actual travel time may vary across regions in the country.

^c^Wait time and servicing time combined represent the total time that a patient spends at the facility from the time that they arrive at the facility to the time that they leave. Wait time from the time that patient arrive to the time that they first interact with a health worker is based on reports from patients in focus groups and all other wait and servicing times are based on observations.

^d^For patients enrolled in the FTR model, the wait and servicing time represents the average time assuming that in a year half of their visits are FTR refills and half are full consultations.

^e^For CAG members, facility-based visit time was observed. The time for community-level meetings is estimated based on input from district staff. This estimate is based on the assumption the CAG meets at the community level before and after each monthly facility appointment. Though actual times were not observed, these community meetings were estimated by district staff to take about one hour each and two meetings take place every month (before and after the clinic visit).

We also used data on patient servicing time to develop unit cost estimates for each model of care and to determine scale up costs. The analysis compared the unit cost of ART service delivery under the MMS, FTR and CAG models to a modelled baseline, which represented costs for an eligible patient making monthly visits and not enrolled in any of the differentiated models of care (Table 9). MMS, FTRs and CAGs all reduced average ART unit costs by approximately 10% but unit costs across the three differentiated models of care were shown to be similar.

**Table 9.** Total ART costs per patient per year, by model of care (for eligible patients only)

Cost category	Modelled baseline	MMS	FTRs	CAGs
**Service delivery costs**	$23.17	$9.25	$8.57	$10.14
Personnel	$14.57	$5.05	$3.81	$3.80
Training	$2.00	$2.00	$2.07	$2.08
Additional supervision for model of care	$0.00	$0.00	$0.49	$3.16
Running costs	$6.60	$2.20	$2.20	$1.10
**Commodity costs**	$112.16	$112.16	$112.16	$112.16
ARVs	$94.45	$94.45	$94.45	$94.45
Laboratory tests	$12.47	$12.47	$12.47	$12.47
Medications for OIs	$5.24	$5.24	$5.24	$5.24
**Total**	$135.33	$121.41	$120.73	$122.30

Unit costs included commodities (ARVs, cotrimoxazole and lab tests) and service delivery costs (personnel, supervision, training and running costs). Commodity costs were estimated from the latest procurement costs in Malawi and accounted for over 92% of total unit costs. These costs were constant across models because the type or quantity of commodities needed is not expected to change based on the model of differentiated care. Instead, models of differentiated care primarily drive changes in ART service delivery costs.

All three models of differentiated care reduced total service delivery costs compared to using none of the models. The MMS model reduces personnel costs by decreasing the number of facility visits for stable ART patients, and therefore the personnel time required to provide care. Patients enrolled in FTRs also visit the facility four times a year like MMS patients, but personnel costs are further reduced by the FTR model because ART refill services are shifted to the HSA, which has a lower compensation level than a clinician. Despite additional costs of training and costs of supervision required for the FTR model as it is currently being executed, the service delivery costs were found to be 7% lower than for the MMS model. Service delivery costs for the CAGs were slightly higher than both the MMS and FTR models. Although the average CAG patient visits the facility less times per year than an MMS or FTR patient, visits are longer due to the volume of patients whose medications are being filled, requiring more personnel time. Additionally, the cost of supervision specific to the CAG model accounts for 30% of total service delivery costs (although only 2% of total unit costs). This includes quarterly HSA-driven supervision of each CAG group. If the CAG model were to be scaled up, these costs may decrease over time as some supervisory roles move from MSF staff to the government given current MOH compensation scales.

## Discussion

Differentiated care can generate efficiencies and improvements in overall quality in the ART service delivery system. MMS is already the standard of care throughout Malawi, and has led to significant reductions in the cost of ART for facilities and patients. However our findings suggest that there is potential for improvement in the implementation of patient differentiation for MMS. There are eligible patients not receiving MMS and ineligible patients who are receiving MMS. Increasing the accuracy of patient differentiation and actively enrolling eligible individuals in MMS will maximize the efficiency benefits of MMS for patients and the health system and may improve quality of care. These results have suggested that gaps may exist in implementation of even simple models of differentiated care, and as such this field would benefit from exploring ways to generate evidence on implementation strength to monitor the models [12].

This study indicated that the FTR and CAGs models do not offer opportunities for further significant reductions in costs beyond those already realized by the MMS model, likely due to the extent to which task shifting and streamlining of services has already occurred in Malawi. While not the focus of this study, these models may offer other benefits beyond system or patient cost savings, including benefits for patient health outcomes. For example, observational studies have suggested that these models are associated with improved patient outcomes. Using data from Chiradzulu District, MSF has reported that rates of death and loss to follow-up were higher among eligible patients that never enrolled in FTR or enrolled late compared to the eligible patients that did enrol in the program [9]. Similarly, research from Mozambique [13], Lesotho [14], and Swaziland [15] indicates that patients enrolled in CAGs have improved retention compared to those not enrolled. This study did not investigate potential differences in patient outcomes resulting from the models, and it should be noted that improvements in outcomes such as viral suppression and adherence may actually serve to reduce long-term costs to the health system [16]. Future research should rigorously assess the impact of these models on patient outcomes and consider these outcomes as a part of more comprehensive costing models.

Beyond benefits to patient outcomes, these models may offer advantages in operational efficiency and patient and provider satisfaction. FTRs may help to improve efficiency in sites where nursing and medical staff are overburdened but lower level cadres that are able to dispense ARVs are available, but as noted by previous research in Malawi, HSAs require significant training and supervision to be qualified to provide high-quality services [17]. Furthermore, as the roles of HSAs and similar cadres expand, there may be a risk of overburdening these cadres as well [18]. Similarly, CAGs may increase social support among patients as well as the accessibility of services for patients in very remote areas, as suggested by research on a similar model in Mozambique [19]. At the same time, the reasons for low uptake of the CAG model in Malawi are not clear. The low uptake may indicate the presence of some implementation challenges which will need to be explored through further research.

These results do suggest that decisions about which models should be offered in a given facility or district should be taken with consideration for the context and the range of potential benefits that a model may offer to facilities that choose to offer them and the patient population that will have access to them. In addition to the context, each specific model should be examined since differentiated care reflects a wide range of programmatic models, which are likely to have varying costs, benefits, and challenges. The study used a mixed methods approach to assess a wide range of characteristics, patterns, trends, and opinions. Wait and service time data collection along with costing data provided real-world application of these findings to assure that the policy implications were relevant. However, the findings of this process evaluation should also be considered in light of several important limitations. The study was designed as a purposefully selected sample to ensure representation of a range of facility types, but was not a random sample of facilities. As such, the inference from this evaluation is limited to the specific sample collected. Additionally, it is important to note that these findings may not be generalizable outside of the Malawian context. The costs of differentiated models of care are largely influenced by the personnel cadres engaged in the models and the national compensation structures in a country and in previous costing studies, Malawi’s national compensation structures are lower than other countries in the region [11]. As a result, cost differences may be greater elsewhere in the region. Finally, this paper focuses on differentiation accuracy, as a key implementation challenge, and on the costs of the models, but the range of potential benefits of these models, in addition to costs, should be considered comprehensively by policy makers.

In order to categorize individuals as active and eligible for specific models of care, some assumptions were applied given the common occurrence of missing data. If data were missing on particular characteristics, we assumed that patients were eligible based on that characteristic. As such, all patients were assumed eligible unless indicated otherwise within the data. While the reasoning for this assumption was sound based on follow-up conversations with health care workers, the assumption also may have led to a misclassification of individuals in some cases. However, there is no evidence that this misclassification would have been differential across the three models of care assessed. Finally, to assess patient adherence, we collected data on the number of doses missed as of the last visit because this is the primary method used to determine patient adherence in facilities in Malawi; however, upon inspection of this data and follow-up inquiries at health facilities, we determined that the data was too inconsistent to be utilized for purposes of this study. In particular, the methods for recording pill count varied greatly across health workers, facilities and record types. Excluding this data from the analysis likely led to the misclassification of some ineligible patients as eligible in our records. However, we believe this misclassification would have been non-differential and all associations are likely conservative. Viral load, which was assessed in this study, is also an objective measure of adherence [20], and patients with poor adherence were likely to be classified as ineligible based on their viral load. Future work should investigate how adherence data may alter these findings.

## Conclusions

MMS is widely implemented in Malawi and has already yielded efficiencies in HIV service delivery for both patients and the health system that are already helping Malawi achieve its national HIV treatment goals; however, optimal implementation of this model will require improvements to the accuracy of patient differentiation. Patient differentiation can be improved through development of more clear guidelines, alignment of registers with eligibility criteria, staff mentorship, standardized use of more objective adherence measures, and especially expanded access to viral load testing. While scale-up of FTRs and CAGs may not produce substantial cost savings in Malawi if implemented as observed in this study, these models may offer other benefits to patients and could be appropriate for some facility contexts or patients. Further, these models may be adapted to produce cost savings. It is also important to note that cost savings from these models may be possible in other countries depending on salary scales and existing level of task shifting. There may also be other models of differentiated care that are not yet widely available in Malawi that could generate benefits. On-going monitoring is needed to understand and continually improve implementation of these models and should include large-scale assessments of the ability of countries like Malawi to use their resources as efficiently as possible to provide high quality care.

## Notes

To access the supplementary material to this article please see Supplementary Files under Article Tools online.

## References

[1] Malawi Ministry of Health Integrated HIV program report: January-March 2016. Lilongwe: Government of Malawi; 2016.

[2] World Health Organization Consolidated guidelines on HIV prevention, diagnosis, treatment and care for key populations. Geneva WHO; 2016 Available from: http://apps.who.int/iris/bitstream/10665/246200/1/9789241511124-eng.pdf 27559558

[3] Malawi Ministry of Health Clinical management of HIV in children and adults. 3rd ed. Lilongwe: Government of Malawi; 2016.

[4] UNAIDS How AIDS changed everything, MDG 6: 15 years, 15 lessons of hope from the AIDS response. Geneva: UNAIDS; 2015 Available from: http://www.unaids.org/en/resources/documents/2015/MDG6_15years-15lessonsfromtheAIDSresponse

[5] Kieffer MP, Mattingly M, Giphart A, van de Ven R, Chouraya C, Walakira M, et al Lessons learned from early implementation of option B+: the Elizabeth Glaser Pediatric AIDS Foundation experience in 11 African countries. J Acquir Immune Defic Syndr. 2014;67(Suppl 4):S188–50. 25436817 10.1097/QAI.0000000000000372 PMC4251909

[6] Global Fund to Fight AIDS, TB and Malaria A toolkit for health facilities: differentiated care for HIV and tuberculosis. Geneva: GFATM; 2015.

[7] International AIDS Society (IAS) Differentiated care for HIV: a decision framework for antiretroviral. 2016 Available from:http://www.differentiatedcare.org/Portals/0/adam/Content/yS6M-GKB5EWs_uTBHk1C1Q/File/Decision%20Framework.pdf

[8] Duncombe C, Rosenblum S, Hellmann N, Holmes C, Wilkinson L, Biot M, et al Reframing HIV care: putting people at the centre of antiretroviral delivery. Trop Med Int Health. 2015;20(4):430–47. 25583302 10.1111/tmi.12460 PMC4670701

[9] Cawley C, Nicholas S, Szumilin E, Perry S, Amoros Quiles I, Masiku C, et al Six-monthly appointments as a strategy for stable antiretroviral therapy patients: evidence of its effectiveness from seven years of experience in a Medecins Sans Frontieres supported programme in Chiradzulu district, Malawi AIDS. Paper presented at: 21st International AIDS Conference; 2016 7 18-22; Durban.

[10] Suresh K, Chandrashekara S. Sample size estimation and power analysis for clinical research studies. J Hum Reprod Sci. 2012;5(1):7–13. 22870008 10.4103/0974-1208.97779 PMC3409926

[11] Tagar E, Sundaram M, Condliffe K, Matatiyo B, Chimbwandira F, Chilima B, et al Multi-country analysis of treatment costs for HIV/AIDS (MATCH): facility-level ART unit cost analysis in Ethiopia, Malawi, Rwanda, South Africa and Zambia. PLoS One. 2014;9(11):e108304. 25389777 10.1371/journal.pone.0108304 PMC4229087

[12] Hargreaves JR, Goodman C, Davey C, Willey BA, Avan BI, Schellenberg JR Measuring implementation strength: lessons from the evaluation of public health strategies in low- and middle-income settings. Health Policy Plan. 2016 9;31(7):860–67. 26965038 10.1093/heapol/czw001 PMC4977426

[13] Jobarteh K, Shiraishi RW, Malimane I, Samo Gudo P, Decroo T, Auld AF, et al Community ART support groups in Mozambique: the potential of patients as partners in care. PLoS One. 2016 12 1;11(12):e0166444. 27907084 10.1371/journal.pone.0166444 PMC5132187

[14] Vandendyck M, Motsamai M, Mubanga M, Makhakhe S, Tunggal S, Jonckheree S, et al Community-based ART resulted in excellent retention and can leverage community empowerment in rural Lesotho, a mixed method study. HIV/AIDS Res Treat Open J. 2015;2(2):44–50.

[15] Pasipamire L, Kerschberger B, Zabsonre I, Ndlovu S, Sibanda G, Mamba S, et al Implementation of combination ART refills models in rural Swaziland. Paper presented at: International AIDS Conference; 2016 7 18-22; Durban.

[16] Jain V, Chang W, Byonanebye DM, Owaraganise A, Twinomuhwezi E, Amanyire G, et al Estimated costs for delivery of HIV antiretroviral therapy to individuals with CD4+ T-cell counts >350 cells/uL in Rural Uganda. PLoS One. 2015 12 3;10(12):e0143433. 26632823 10.1371/journal.pone.0143433 PMC4669141

[17] Tweya H, Feldacker C, Ben-Smith A, Weigel R, Boxshall M, Phiri S, et al ‘Task shifting’ in an antiretroviral clinic in Malawi: can health surveillance assistants manage patients safely? Public Health Action. 2012 12 21;2(4):178–80. 26392980 10.5588/pha.12.0018 PMC4463055

[18] Smith S, Deveridge A, Berman J, Negin J, Mwambene N, Chingaipe E, et al Task-shifting and prioritization: a situational analysis examining the role and experiences of community health workers in Malawi. Hum Resour Health. 2014 5;2(12):24. 10.1186/1478-4491-12-24 PMC4014628 24885454

[19] Rasschaert F, Telfer B, Lessitala F, Decroo T, Remartinez D, Biot M, et al A qualitative assessment of a community antiretroviral therapy group model in Tete, Mozambique. PLoS One. 2014 3 20;9(3):e91544. 24651523 10.1371/journal.pone.0091544 PMC3961261

[20] Working Group on Modelling of Antiretroviral Therapy Monitoring Strategies in Sub-Saharan Africa Sustainable HIV treatment in Africa through viral-load-informed differentiated care. Nature. 2015;528(7580):S68–76. 26633768 10.1038/nature16046 PMC4932825

